# AI-Enhanced Quantitative IHC Analysis for Prognostic Stratification in Marginal Zone Lymphoma: Development of a Revised MZL-IPI Model

**DOI:** 10.3390/diagnostics16101456

**Published:** 2026-05-11

**Authors:** Qingyang Zhang, Zeyu Deng, Wenzhe Yan, Hongkai Zhu, Zizhu Tian, Yi Jiang, Hongling Peng

**Affiliations:** 1 Department of Hematology, The Second Xiangya Hospital of Central South University, Changsha 410011, China; 2 Department of Pathology, The Second Xiangya Hospital of Central South University, Changsha 410011, China; 3 Department of Pathology, Guilin Hospital of the Second Xiangya Hospital CSU, Guilin 541002, China; 4 Hunan Engineering Research Center of Cell Immunotherapy for Hematopoietic Malignancies, Changsha 410011, China

**Keywords:** marginal zone lymphoma, artificial intelligence, immunohistochemistry quantification, prognostic biomarkers, revised MZL-IPI model

## Abstract

**Objectives**: Marginal zone lymphoma (MZL) is a heterogeneous indolent B-cell lymphoma, and current clinical prognostic systems remain limited in identifying transformation risk and refining risk stratification. This study aimed to evaluate the prognostic relevance of artificial intelligence (AI)-quantified immunohistochemical (IHC) markers in MZL and to explore a revised MZL-IPI model. **Methods**: We retrospectively analyzed 146 patients with pathologically confirmed MZL treated at the Second Xiangya Hospital of Central South University from January 2015 to June 2022. Among them, 111 patients had digitized IHC slides available for AI-assisted quantitative analysis. AI-quantified IHC marker expression was assessed in relation to clinical features, histologic transformation, and survival outcomes. Prognosis-related markers were dichotomized using optimal cut-off values. Survival differences were evaluated using the log-rank test, independent prognostic factors were identified by multivariable Cox regression, and model performance was assessed by cross-validation. **Results**: Age, B symptoms, hypertension, diabetes mellitus, and gastric involvement were associated with selected IHC parameters. CD3 was independently associated with histologic transformation, with expression below 25.60% indicating higher transformation risk. High CD21 expression independently predicted favorable overall survival (OS), whereas high CD3 expression was associated with inferior progression-free survival (PFS). Incorporating CD21 into the MZL International Prognostic Index (MZL-IPI) improved OS prediction in this cohort. **Conclusions**: AI-assisted quantitative IHC analysis may provide complementary prognostic information in MZL. The CD21-revised MZL-IPI represents an exploratory framework for integrating AI-derived tissue biomarkers with clinical risk stratification, but external multicenter validation is required before clinical application.

## 1. Introduction

Marginal zone lymphoma (MZL) is the second most common subtype of indolent B-cell non-Hodgkin lymphoma (NHL), accounting for approximately 5–15% of all NHL cases. According to the World Health Organization (WHO) classification, MZL is further categorized into mucosa-associated lymphoid tissue lymphoma (MALT lymphoma), nodal marginal zone lymphoma (NMZL), and splenic marginal zone lymphoma (SMZL), which account for 50–70%, approximately 20%, and approximately 10% of MZL cases, respectively [1]. MZL originates from marginal-zone B cells expressing clonal immunoglobulin and is closely associated with chronic bacterial or viral infection, autoimmune disease, and persistent antigenic stimulation. These factors can induce sustained inflammation and immune activation, thereby promoting B-cell expansion, genetic alterations, and malignant clonal evolution [2]. The diagnosis of MZL primarily relies on pathological biopsy combined with morphologic, immunophenotypic, and genetic analyses. Typical immunohistochemical features include positivity for CD19, CD20, and CD22 and negativity for CD5, CD10, and CD23.

Beyond conventional morphologic and immunophenotypic assessment, several emerging technologies have increasingly been applied to the diagnosis and biological evaluation of MZL in recent years [3]. Fluorescence in situ hybridization and other cytogenetic methods can detect recurrent chromosomal abnormalities; immunoglobulin gene rearrangement analysis can help determine B-cell clonality [4]; and next-generation sequencing can identify MZL-associated genetic alterations and pathway abnormalities, thereby complementing conventional pathological diagnosis and assisting in the differential diagnosis of small B-cell lymphomas [5]. In addition, multiplex immunohistochemistry and spatial omics technologies can provide more refined information regarding immune-cell composition, tissue architecture, and interactions between tumor cells and the microenvironment [6]. These advances suggest that MZL diagnosis is moving toward an integrated model that combines morphology, immunophenotyping, molecular genetics, and quantitative assessment of the tumor microenvironment.

At the same time, artificial intelligence (AI) has rapidly advanced in histopathology, providing new tools for lymphoma diagnosis, quantitative biomarker assessment, and prognostic stratification [7]. Through whole-slide imaging and computational image analysis, digital pathology enables objective and reproducible evaluation of tissue morphology and IHC features [8,9]. AI models have been applied to tissue segmentation, lesion detection, tumor microenvironment assessment [10], and lymphoma subtype classification, and several studies have reported diagnostic performance comparable to that of expert pathologists [11,12,13]. In particular, AI-assisted IHC quantification can reduce interobserver variability [14] and provide continuous, reproducible measurements of biomarker expression, which may offer complementary information for lymphoma risk assessment [15,16].

Despite advances in molecular biology and cytogenetics, current clinical scoring systems, such as the MZL-IPI and Ann Arbor staging, remain limited in predicting prognosis in MZL, particularly in identifying high-risk patients and evaluating the risk of histologic transformation. Therefore, prognostic models that integrate clinical parameters, histological features, and quantitative biomarkers are needed to improve risk stratification in MZL.

In this context, the present study used AI to quantify IHC markers and evaluated their associations with clinical characteristics, histologic transformation, and survival outcomes. We further explored whether AI-derived IHC parameters could complement the existing MZL-IPI model and provide a potential framework for MZL risk stratification after external validation.

## 2. Materials and Methods

### 2.1. Study Population

**Figure 1:**
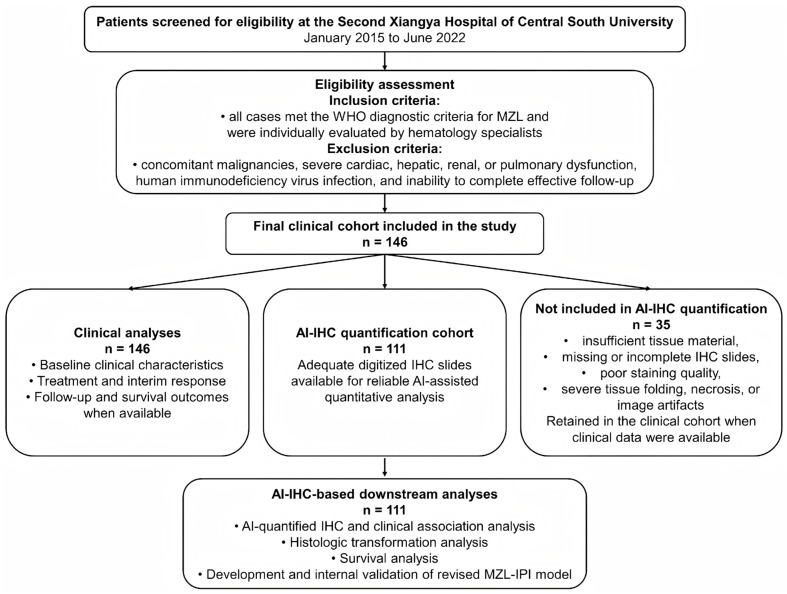
Patient selection and study design flowchart. This flowchart summarizes patient screening, inclusion in the clinical cohort, AI-assisted IHC quantification, and subsequent statistical analyses. A total of 146 patients with MZL were included in the clinical analysis cohort; among them, 111 had digitized IHC slides available for AI-assisted quantitative analysis, whereas 35 were not included in AI-IHC quantification because of insufficient tissue material, missing or incomplete IHC slides, poor staining quality, severe tissue folding, necrosis, or image artifacts. Subsequent analyses included clinical characteristic analysis, association analysis between AI-quantified IHC markers and clinical features, histologic transformation analysis, survival analysis, and construction and internal validation of the revised MZL-IPI model.

This retrospective study collected clinical and laboratory data from 146 patients with pathologically confirmed MZL who were treated at the Second Xiangya Hospital of Central South University between January 2015 and June 2022. The inclusion criteria were as follows: all cases met the WHO diagnostic criteria for MZL and were individually evaluated by hematology specialists. Exclusion criteria included concomitant malignancies, severe cardiac, hepatic, renal, or pulmonary dysfunction, human immunodeficiency virus infection, and inability to complete effective follow-up. The collected clinical variables included sex, age, primary site, histologic subtype, transformation to diffuse large B-cell lymphoma (DLBCL), Ann Arbor stage, ECOG performance status, MZL-IPI score, B symptoms, hypertension, diabetes mellitus, hepatitis B virus infection, *Helicobacter pylori* infection, complete blood count parameters (white blood cells, hemoglobin, and platelets), serum albumin, lactate dehydrogenase (LDH), and *β*2-microglobulin (*β*2-MG). The patient selection and study workflow are shown in Figure 1.

### 2.2. AI-Assisted Immunohistochemical Analysis

All pathological specimens were obtained from surgical or endoscopic biopsies, fixed in 10% formalin, embedded in paraffin, sectioned, and stained with hematoxylin and eosin (H&E) and IHC. Routine IHC markers included Bcl-2, Bcl-6, CD3, CD5, CD10, CD20, CD21, CD23, CD30, CD79a, CyclinD1, c-MYC, Ki-67, Mum1, and PAX5. All diagnoses were confirmed by experienced pathologists based on tissue morphology and IHC findings.

AI analysis was performed using an Intelligent Pathology Stereoscopic Analyzer (LSD-PSA-024C; Changsha Lansi Intelligent Technology Co., Ltd., Changsha, China). The system scanned H&E and IHC slides in whole-slide mode and selected regions with high tumor-cell content and minimal tissue distortion, fibrosis, necrosis, or artifacts for analysis. Among the 146 patients in the clinical cohort, 111 had digitized IHC slides available for AI-assisted quantitative analysis. The remaining 35 patients were not included in the AI-IHC quantification cohort because of insufficient tissue material, missing or incomplete IHC slides, poor staining quality, severe tissue folding, necrosis, or image artifacts that precluded reliable AI-based segmentation and quantification. When appropriate clinical data were available, these patients were retained in the overall clinical cohort but were excluded from analyses requiring AI-quantified IHC parameters.

IHC slides were digitized into high-resolution images and automatically quantified using intelligent stereoscopic analysis software (version 1.0) based on deep convolutional neural networks. The analysis workflow included the following steps: (1) cell recognition on H&E slides, in which cell contours and positions were identified based on morphologic features; (2) cross-slide image registration to match cell positions between H&E and IHC slides; (3) color deconvolution and normalization; (4) cell segmentation using a watershed algorithm; (5) extraction of IHC-positive regions through automatic thresholding and morphological processing; and (6) calculation of the proportions of positive and negative cells and output of quantitative IHC metrics.

For the *r*-th region of interest (ROI), the IHC positivity rate was defined as:

$$P_{r}=\frac{N_{positive,r}}{N_{total,r}}\times 100\%$$

where $N_{positive,r}$ represents the number of cells classified as positive in that ROI, and $N_{total,r}$ represents the total number of cells identified in that ROI. For an individual cell i, the positivity score was defined as:

$$q_{i}=\frac{A_{positive,i}}{A_{cell,i}}$$

where $A_{positive,i}$ indicates the IHC-positive pixel area within the cellular or nuclear region, and $A_{cell,i}$ indicates the total segmented area of the cell or nucleus. When $q_{i}\geq \alpha$, the cell was classified as positive, where α represents the positivity threshold determined by the system’s automatic thresholding algorithm.

Because staining intensity may be affected by tissue fixation, processing procedures, section thickness, and staining conditions, the present study used the proportion of positive cells rather than staining intensity as the primary quantitative output. The system included a built-in quality-control workflow to assess background staining, signal-to-noise ratio, tissue folding, necrosis, and artifact-prone regions before analysis. The final AI-quantified IHC metrics were automatically exported for subsequent statistical analysis. The device has obtained six Chinese invention patents (ZL202110421778.X, ZL2020115131625.2, ZL202110158033.9, ZL202111558334.7, ZL202011554677.1, and ZL202011301331.0) and was certified by the National Medical Products Administration (NMPA), with the registration certificate issued on 9 November 2022 (XZZ 20222222047) [17].

### 2.3. Follow-Up and Survival Outcomes

Survival information was collected through outpatient visits, inpatient follow-up, and telephone interviews through 15 May 2023. Interim treatment response was assessed after 2–4 treatment cycles using whole-body CT, PET/CT, or gastrointestinal endoscopy. According to WHO criteria, treatment responses were classified as favorable response, including complete remission (CR) or partial remission (PR), or unfavorable response, including stable disease (SD) or progressive disease (PD). Survival endpoints included progression-free survival (PFS), defined as the time from diagnosis to first relapse, progression, or death, and overall survival (OS), defined as the time from diagnosis to death or last follow-up.

### 2.4. Statistical Analysis

All statistical analyses were performed using R software (version 4.3). Quantitative variables are presented as the mean ± standard error or median (interquartile range), and categorical variables are presented as counts and percentages. Between-group comparisons were performed using the *χ*^2^ test or Fisher’s exact test for categorical variables, the independent-samples *t* test for normally distributed continuous variables, the Mann–Whitney U test for nonnormally distributed continuous variables, and analysis of variance or the Kruskal–Wallis test for multiple-group comparisons. Correlations were assessed using Pearson correlation coefficients.

Cut-off values were determined according to the endpoint and analytical purpose. For histologic transformation, which was treated as a binary outcome, receiver operating characteristic (ROC) curve analysis was used to evaluate predictive performance, and the optimal cut-off value was determined by maximizing the Youden index, calculated as sensitivity + specificity − 1. For Kaplan–Meier survival analyses of individual AI-IHC markers, cut-off values were estimated using the surv_cutpoint function in the R package survminer to identify thresholds that maximized survival differences. Multivariable Cox regression analysis was used to identify independent prognostic factors, and model performance was evaluated using the concordance index (C-index).

For construction of the revised MZL-IPI model, candidate cut-off values for CD21 and CD3 were evaluated within an internal repeated random resampling and three-fold cross-validation framework. In each random split, approximately two-thirds of the samples were assigned to the training set and one-third to the validation set. IHC marker cut-off values were first determined in the training set and then applied unchanged to the corresponding validation set. The revised model was defined as follows: revised MZL-IPI = MZL-IPI + IHC score, and model performance was assessed using the C-index in the validation set. This procedure was repeated across 100,000 random training/validation splits, and the final cut-off values were selected according to the overall improvement in validation-set C-index for the revised model compared with the original MZL-IPI. Multiple testing was adjusted using the Benjamini–Hochberg false discovery rate method. Visualizations were generated using ggplot2 package version 4.0.1, and *p* < 0.05 was considered statistically significant.

## 3. Results

The overall workflow of patient screening, enrollment, AI-assisted IHC quantification, and subsequent statistical analysis is shown in Figure 1. Patients with pathologically confirmed MZL treated at the Second Xiangya Hospital of Central South University between January 2015 and June 2022 were retrospectively screened. According to predefined inclusion and exclusion criteria, 146 patients were included in the clinical analysis cohort, of whom 111 had digitized IHC slides available for AI-assisted quantitative analysis.

### 3.1. Clinical Characteristics and Distribution of Quantitative IHC Markers

A total of 146 patients with MZL were included, including 83 men (56.85%) and 48 patients aged ≥ 60 years (32.88%). The mean age was 55.17 years (range, 19–83 years). The primary disease sites were mainly visceral extranodal sites, with gastric involvement observed in 41 patients (28.08%). Histologic subtypes included MALT lymphoma in 81.51% (119/146), SMZL in 13.01% (19/146), and NMZL in 4.79% (7/146), with one additional case classified as MZL not otherwise specified (MZL-NOS). Histologic transformation occurred in 21 patients (14.38%), including 20 cases originating from MALT lymphoma and one case from NMZL.

Because treatment strategies for indolent lymphoma are heterogeneous, patients received observation, surgery, radiotherapy, chemotherapy, immunochemotherapy, or combinations thereof. Chemotherapy was administered to 86.99% of patients (127/146), including CHOP in 80.32% (102/127) and BR in 12.60% (16/127). Radiotherapy and surgery were performed in 15.07% and 30.14% of patients, respectively, and rituximab was used in 80.82%. Regarding clinical symptoms and comorbidities, B symptoms were present in 24.66% of patients, and hypertension, diabetes mellitus, HBV infection, and *H. pylori* infection were present in 17.81%, 8.22%, 20.55%, and 18.49%, respectively.

Baseline laboratory data showed a mean white blood cell count of 6.26 × 10^9^/L (range, 1.72–27.52 × 10^9^/L), hemoglobin level of 119.18 g/L (range, 50–241 g/L), platelet count of 226.68 × 10^9^/L (range, 9–559 × 10^9^/L), *β*2-MG level of 3.14 mg/L (range, 0.05–13.31 mg/L), LDH level of 251.37 U/L (range, 91.1–3467.5 U/L), and albumin level of 37.78 g/L (range, 18.7–48.1 g/L). ECOG performance status ≥ 2 was observed in 26.02% of patients, Ann Arbor stage III/IV disease in 56.84%, and MZL-IPI > 2 in 34.25%. The CR/PR rate at interim response assessment was 74.66% (109/146) (Supplementary Tables S1 and S2).

### 3.2. AI-Quantified IHC Results and Associations with Clinical Characteristics

**Figure 2:**
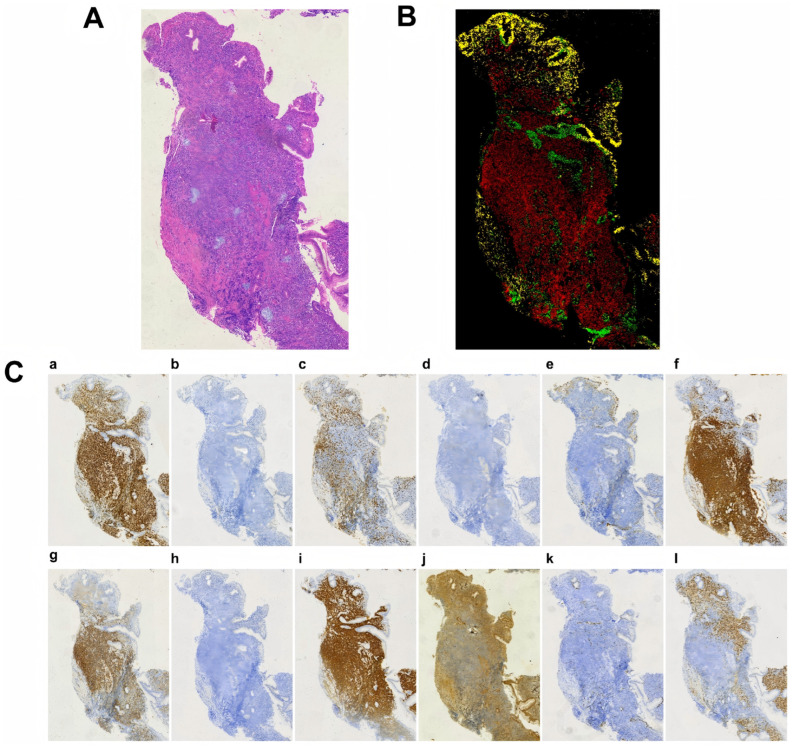
Representative digitized immunohistochemical (IHC) slide images. (**A**) H&E-stained image; (**B**) pseudocolor composite image of Bcl-2 and CD20, in which red indicates Bcl-2^+^/CD20^+^, green indicates Bcl-2^+^/CD20^−^, and yellow indicates Bcl-2^−^/CD20^+^; (**C**) individual IHC-stained images, including (**a**) Bcl-2, (**b**) Bcl-6, (**c**) CD3, (**d**) CD5, (**e**) CD10, (**f**) CD20, (**g**) CD21, (**h**) CD30, (**i**) CD79a, (**j**) c-MYC, (**k**) Ki-67, and (**l**) Mum1. All images were acquired at 80× magnification.

Among the 111 patients with MZL, 15 IHC markers (Bcl-2, Bcl-6, CD3, CD5, CD10, CD20, CD21, CD23, CD30, CD79a, c-MYC, Ki-67, Mum1, PAX5, and CyclinD1) were quantitatively analyzed using AI, and representative digitized images are shown in Figure 2. To ensure analytical reliability, markers with sample sizes < 30 were excluded, leaving 13 IHC markers for subsequent analyses (Supplementary Table S3). These markers covered T- and B-cell lineages and pathways related to cell proliferation, apoptosis, and differentiation.

The mean values and 95% confidence intervals (CIs) for each IHC marker are shown in Supplementary Figure S1. CD20 and CD79a showed the highest expression levels, consistent with their roles as B-cell markers. Stratified analyses of clinical characteristics showed no significant differences in IHC expression according to sex, MZL subtype, or interim treatment response (Supplementary Figure S2A–D). CD20 expression was increased in patients aged ≥ 60 years; CyclinD1 was upregulated and PAX5 was downregulated in patients with B symptoms; CD3 and CD20 were upregulated in patients with hypertension; CyclinD1 was downregulated and PAX5 was upregulated in patients with diabetes mellitus; HBV infection was not significantly associated with any IHC marker; CD5 was downregulated in the HP-infected group; and CD21 and Mum1 were upregulated, whereas Bcl-2, CD20, and CD79a were downregulated, in patients with gastric involvement (Supplementary Figure S2E–J). These results indicate that AI-quantified IHC markers are associated with selected clinical characteristics.

### 3.3. Association Between IHC Markers and Histologic Transformation

**Figure 3:**
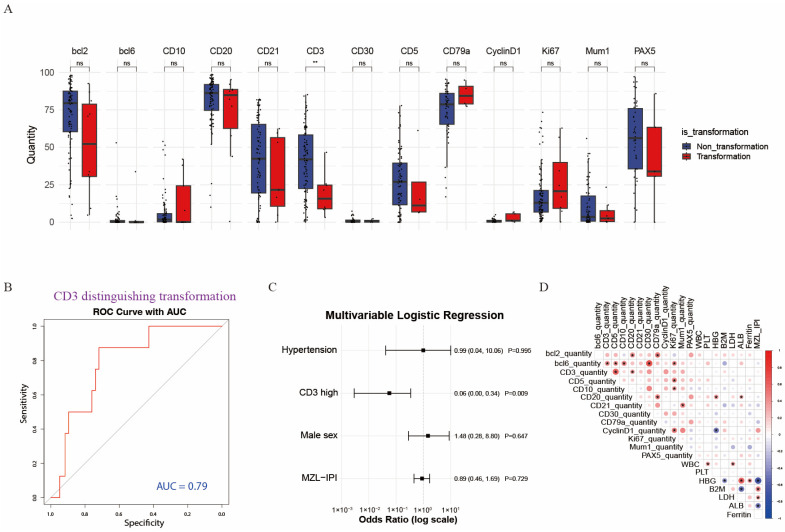
Associations of AI-quantified IHC markers with histologic transformation and continuous clinical variables. (**A**) Boxplots showing differences in the expression of 13 AI-quantified IHC markers between non-transformed and transformed cases, with significantly lower CD3 expression in the transformed group; (**B**) ROC curve showing the discriminatory performance of CD3 for histologic transformation, with an AUC of 0.79; (**C**) multivariable logistic regression forest plot showing that CD3 remained an independent protective factor associated with histologic transformation after adjustment for confounders, with high expression significantly reducing the risk of transformation; (**D**) correlation heatmap showing associations between AI-quantified IHC markers and continuous clinical variables. Deeper red indicates stronger positive correlations, and deeper blue indicates stronger negative correlations. In panel A, *ns* indicates not significant, and ** indicates *p* < 0.01. In panel D, * indicates *p* < 0.05.

Histologic transformation occurred in 21 patients. Compared with the non-transformed group, the transformed group showed significantly lower CD3 expression (Figure 3A). ROC analysis showed that CD3 predicted transformation with an area under the curve (AUC) of 0.79, and the optimal cut-off value determined by the Youden index was 25.60% (Figure 3B). This cut-off was specifically determined for the binary endpoint of histologic transformation and was therefore used only for transformation-risk analysis. Multivariable logistic regression showed that, after excluding redundant variables already included in the MZL-IPI, CD3 remained independently associated with transformation; high CD3 expression significantly reduced the risk of transformation (OR = 0.06, 95% CI 0.00–0.36, *p* = 0.01) (Figure 3C).

### 3.4. Correlations Between IHC Markers and Continuous Variables

Correlation analysis showed that CD3 was positively correlated with CD5 and CD20. Bcl-2 was positively correlated with CD3 and CD20 and negatively correlated with Bcl-6 and Ki-67. MZL-IPI was positively correlated with CD30, WBC, and β2-MG and negatively correlated with CD21, hemoglobin, and albumin. Ki-67 was positively correlated with CD3, CD5, CD10, and CyclinD1 (Figure 3D). These findings are consistent with several known immunobiological features and indirectly support the reliability of AI-based quantification.

### 3.5. Prognostic Associations of IHC Markers

As of 15 May 2023, the longest follow-up duration was 208 months. The mean follow-up for PFS was 42.22 months, with a progression rate of 30.14%; the mean follow-up for OS was 46.65 months, with an overall mortality rate of 11.64% (Supplementary Table S2). Based on the AI-quantified IHC results, we further assessed the associations between individual IHC markers and prognosis.

**Figure 4:**
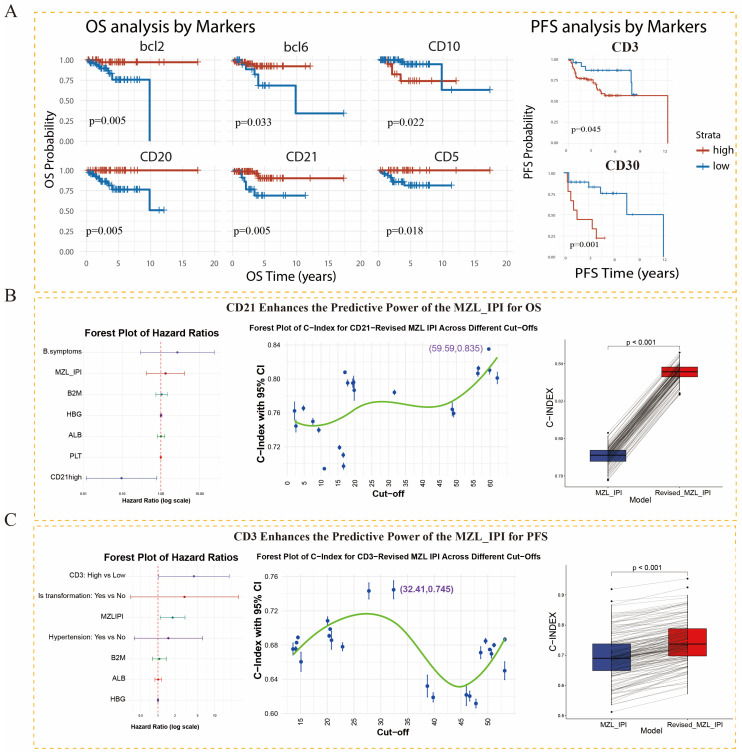
Prognostic analysis of AI-quantified IHC markers and construction of the revised MZL-IPI model. (**A**) Kaplan–Meier survival curves for OS and PFS. In the OS analysis, high expression of Bcl-2, Bcl-6, CD20, CD21, and CD5 was associated with better survival, whereas high CD10 expression was associated with worse survival. In the PFS analysis, high CD3 and CD30 expression was associated with inferior PFS. (**B**,**C**) Each panel consists of three components: the left panel shows multivariable Cox regression forest plots of IHC markers and clinical factors; the middle panel shows cross-validation results, with the *x*-axis indicating the cut-off value and the *y*-axis indicating the C-index and confidence interval; the right panel shows paired comparisons of the C-index between the revised MZL-IPI and original MZL-IPI in the validation set using 100 bins, with the mean value indicated. (**B**) Incorporation of CD21 improved model discrimination for OS prediction. (**C**) Incorporation of CD3 improved model discrimination for PFS prediction.

Patients were divided into high- and low-expression groups according to the optimal cut-off value for each IHC marker. OS analysis showed that high expression of Bcl-2, Bcl-6, CD20, CD21, and CD5 was associated with better OS, whereas high CD10 expression was associated with worse OS. PFS analysis showed that high CD3 and CD30 expression was associated with inferior PFS (Figure 4A).

In independent prognostic analyses, we further accounted for potential confounding effects related to treatment heterogeneity. Treatment modality was decomposed into four variables: chemotherapy, radiotherapy, surgery, and rituximab use. These variables were incorporated as stratification variables in the multivariable Cox regression model to allow different treatment groups to have independent baseline hazard functions. Clinical variables associated with IHC quantitative values or prognosis were also included as confounders.

Multivariable analysis of OS showed that, among the six IHC markers associated with OS, only CD21 retained independent prognostic significance after adjustment for confounders, with high expression indicating better survival (Figure 4B, left). In the PFS multivariable analysis, CD30 was not included because of its limited available sample size (*n* = 36); CD3 remained an independent adverse prognostic factor after adjustment, with the hazard ratio and 95% CI both exceeding 1 (Figure 4C, left).

### 3.6. Optimization of the MZL-IPI Risk Model

Because CD21 and CD3 were identified as independent factors associated with OS and PFS, respectively, we further evaluated whether they could improve the predictive performance of the MZL-IPI. Candidate cut-off values for CD21 and CD3 were assessed within an internal repeated random resampling and three-fold cross-validation framework. In each random split, approximately two-thirds of the samples were assigned to the training set and one-third to the validation set, and model performance was evaluated using the C-index. IHC quantitative values were first dichotomized in the training set according to candidate cut-off values, and an IHC score was then constructed as follows: for CD21, high expression was assigned 0 points because it was associated with better OS, and low expression was assigned 1 point; for CD3, high expression was assigned 1 point because it was associated with worse PFS, and low expression was assigned 0 points. The revised model was defined as follows: revised MZL-IPI = MZL-IPI + IHC score. This procedure was repeated across 100,000 random training/validation splits, and the final cut-off values were determined according to the overall improvement in validation-set C-index for the revised model compared with the original MZL-IPI.

For OS prediction, the optimal CD21 cut-off was 56.53% (Figure 4B, middle). Across all validation sets, the C-index of the revised MZL-IPI was significantly higher than that of the original MZL-IPI (*p* < 0.001) (Figure 4B, right), suggesting that CD21 improved the OS-prediction performance of the MZL-IPI. The model was calculated as follows: revised MZL-IPI = MZL-IPI + CD21 score (CD21 > 56.53% = 0 points; otherwise = 1 point).

For PFS prediction, the optimal CD3 cut-off was 32.41% (Figure 4C, middle), and the revised MZL-IPI also outperformed the original model in the validation sets (*p* < 0.001) (Figure 4C, right). The model was calculated as follows: revised MZL-IPI = MZL-IPI + CD3 score (CD3 > 32.41% = 1 point; otherwise = 0 points). It should be noted that the CD3 cut-off used for histologic transformation analysis differed from the cut-off used for PFS model construction because the two thresholds were derived for different endpoints and analytical purposes. The CD3 threshold of 25.60% was determined by ROC analysis for histologic transformation and was used to distinguish whether histologic transformation occurred, whereas the CD3 threshold of 32.41% was selected within the internal resampling framework to construct a CD3-based revised MZL-IPI for optimizing PFS prediction. Similarly, the CD21 threshold of 56.53% was selected to construct a CD21-based revised MZL-IPI for predicting OS. These IHC marker thresholds were internally optimized in the present cohort and should not be regarded as universal clinical cut-offs; validation in independent multicenter cohorts remains necessary.

**Figure 5:**
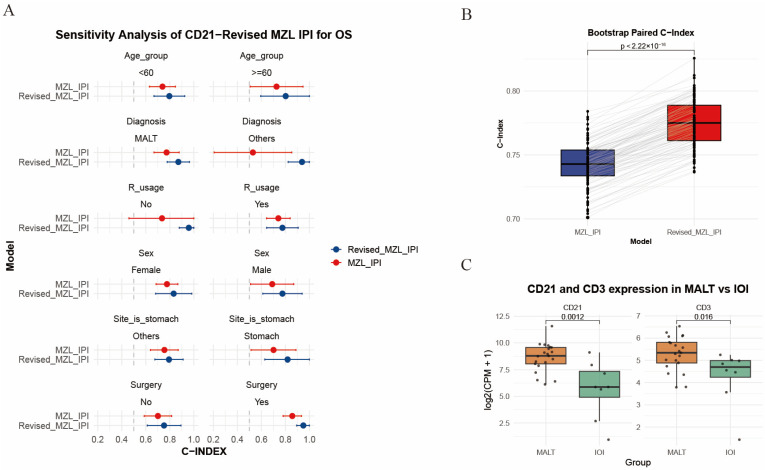
Sensitivity analysis and validation of CD21/CD3 expression in external datasets. (**A**) Forest plot comparing the C-index and confidence intervals of the CD21-revised MZL-IPI and the original MZL-IPI for OS prediction across different clinical strata, including age, diagnostic subtype, rituximab use, sex, primary disease site, and surgery status; (**B**) comparison of the C-index between the CD21-revised MZL-IPI and original MZL-IPI in the homogeneous-treatment subgroup, showing better predictive performance of the CD21-revised model; (**C**) gene expression analysis based on log(CPM + 1) showing that CD21 and CD3 were both upregulated in MALT lymphoma compared with idiopathic orbital inflammation (IOI).

To further evaluate model robustness, we performed sensitivity analyses across subgroups defined by age, sex, treatment modality, and histologic subtype. Because C-index estimation requires adequate sample sizes, subgroups with fewer than 20 patients were excluded. For OS prediction, the CD21-revised MZL-IPI outperformed the original MZL-IPI across subgroups stratified by age, diagnostic subtype, rituximab use, sex, primary disease site, and surgery status (Figure 5A). In a strictly defined homogeneous-treatment subgroup consisting of patients with MALT lymphoma who received rituximab plus chemotherapy without surgery or radiotherapy, bootstrap resampling was performed 3000 times to generate 100 C-index bins. The results also showed better predictive performance for the CD21-revised model (Figure 5B).

In contrast, the CD3-revised MZL-IPI was less stable than the CD21-revised model. Although it outperformed the original model in the cross-validation validation sets, sensitivity analyses showed advantages only in selected subgroups, such as patients aged > 60 years, patients with favorable interim response, and male patients; no obvious improvement was observed in the homogeneous-treatment subgroup (Supplementary Figure S3). Overall, the CD21-revised model showed relatively stable predictive performance, whereas the CD3-revised model may be more susceptible to cohort-specific effects and potential overfitting. As a supplementary observation, we also used idiopathic orbital inflammation (IOI) data from GSE171059 and GSE199517 as controls [18] and found that CD21 and CD3 ex-pression levels were both higher in MALT lymphoma samples than in IOI samples (log(CPM + 1), Figure 5C), suggesting that these markers may be related to immune microenvironmental features of MALT lymphoma, although this finding requires fur-ther validation. All adjusted analysis results are provided in Supplementary Tables S4–S6.

## 4. Discussion

AI-assisted digital pathology is transforming histopathological assessment from traditional qualitative interpretation toward quantitative and reproducible analysis [19]. Digital pathology enables quantitative analysis of pathological sections, including quantification of immunomarkers and prediction of molecular expression patterns [20]. In lymphoma, AI has been used to extract nuclear geometric features in DLBCL with independent prognostic significance [21], assist in malignant lymphoma subtype classification [22], and improve diagnostic accuracy in chronic lymphocytic leukemia by integrating cellular morphology and spatial distribution features [23,24]. Deep learning-based convolutional neural network models have shown favorable performance in diagnosing multiple NHL subtypes, with reported accuracies of 95% [25] or higher [26], although performance remains affected by image quantity, image quality, and model-training strategies [27]. AI has also shown potential in predicting large-cell transformation in indolent B-cell lymphomas [28], optimizing NHL subtype classification [29], and integrating imaging with genomic data to predict treatment response [30].

In this study, we used AI to quantitatively analyze IHC-stained images from patients with MZL and evaluated associations with clinical characteristics, histologic transformation, and survival outcomes. Age, B symptoms, hypertension, diabetes mellitus, and gastric involvement were associated with selected IHC markers, suggesting possible links between clinical phenotypes and pathological expression patterns. Previous studies have demonstrated the value of clinical prognostic models in MZL, including the Italian Lymphoma Intergroup model for SMZL based on hemoglobin, albumin, and LDH [31], and the HPLL model, which incorporates additional clinical variables to improve risk stratification [32]. In addition, H. pylori-associated MALT lymphoma can benefit from eradication therapy, highlighting the importance of environmental and immune factors in MZL pathogenesis [33].

Although MZL generally follows an indolent clinical course, it retains a risk of histologic transformation to DLBCL. We found that CD3 was independently associated with transformation in MZL. CD3 is an essential component of the T-cell receptor complex [34] and participates in TCR signaling and T-cell activation [35]. Previous studies suggest that T-cell help in the setting of chronic inflammation may contribute to MALT lymphoma development [36], and immune-cell interactions within the tumor microenvironment may also influence transformation risk [37]. In the present study, low CD3 expression was associated with a higher risk of transformation, suggesting that reduced T-cell infiltration may reflect impaired immune surveillance or insufficient T-cell-mediated antitumor responses. However, high CD3 expression was also associated with inferior PFS, indicating that the prognostic meaning of CD3 may be context-dependent. Total CD3 expression does not distinguish cytotoxic T cells, helper T cells, regulatory T cells, exhausted T cells, or other T-cell subsets. Therefore, increased CD3 expression does not necessarily represent effective antitumor immunity and may instead reflect chronic antigen stimulation, inflammatory immune activation, immune exhaustion, or enrichment of immunosuppressive T-cell populations. Future studies integrating CD4, CD8, FOXP3, PD-1, and spatial immune analyses are needed to clarify the roles of T-cell subsets in MZL progression.

Correlation analysis further suggested biological relationships among different IHC markers in MZL. CD3 was positively correlated with CD5, and CD5 expression in several lymphomas has been associated with immune tolerance, abnormal regulation of TCR/BCR signaling, and tumor immune escape [38,39,40,41]. Bcl-2 was positively correlated with CD3 and CD20 and negatively correlated with Bcl-6 and Ki-67, consistent with its role in promoting cell survival in indolent lymphomas [42,43,44,45]. The associations of Bcl-6 with CD10 and CD5 reflect its relationship with germinal-center differentiation [46,47]. Ki-67 was associated with several T- and B-cell markers, suggesting that proliferative activity may be related to the composition of the tumor microenvironment. As a B-cell-associated marker, CD20 is important for lymphoma diagnosis and response to rituximab-based therapy [48,49,50,51,52,53].

We also found that CD21 was independently associated with OS and improved the OS-prediction performance of the MZL-IPI model. CD21, also known as complement receptor 2, participates in B-cell activation and complement-mediated immune regulation and is commonly used as a marker of follicular dendritic cell (FDC) networks [54]. In MZL, CD21-positive FDC networks may reflect preservation or remodeling of lymphoid follicular architecture [55]. High CD21 expression was associated with better OS, which may indicate relatively preserved follicular structures, more intact FDC networks, and a more organized immune microenvironment. Conversely, low CD21 expression may reflect disruption of the FDC network, disorganized lymphoid architecture, and remodeling of the tumor microenvironment, which may be associated with poorer survival. Thus, AI-quantified CD21 may capture microenvironmental structural information that is not fully reflected by conventional clinical prognostic scoring systems. Prior studies have shown that tumor-intrinsic CD21 expression can affect recognition of CD19-positive tumor cells by CD19-CAR-T cells and reduce their cytotoxic effects [56]. In addition, an increased proportion of immune checkpoint-positive T cells in the tumor microenvironment has been associated with adverse prognosis [57]. Nevertheless, these biological explanations remain hypothetical. The present study demonstrates associations between AI-quantified CD3/CD21 expression and clinical outcomes but does not establish causality. Functional experiments and spatially resolved immune microenvironment analyses are required to validate these findings.

The revised MZL-IPI constructed using AI-quantified IHC markers showed better prognostic discrimination than the original MZL-IPI in this cohort, especially the CD21-revised MZL-IPI for OS prediction. Sensitivity analyses showed that the CD21-revised model maintained favorable predictive performance across several clinical subgroups and in a homogeneous-treatment subgroup. In contrast, the CD3-revised MZL-IPI showed less stable performance and demonstrated advantages only in selected subgroups, suggesting greater susceptibility to cohort-specific effects and potential overfitting. Therefore, CD21 may represent a more stable AI-quantified IHC marker for complementing clinical prognostic indices, whereas the clinical utility of CD3 requires further validation, particularly through analyses of T-cell subsets and spatial distribution patterns.

Several limitations should be acknowledged. First, this was a retrospective single-center study with a relatively limited sample size. Although 146 patients were included in the clinical cohort, only 111 had slides available for AI-assisted quantitative IHC analysis, and the number of survival events was limited, which may restrict the statistical power and robustness of model development. Second, the AI-derived CD3 and CD21 cut-off values were generated from this cohort using a specific commercial Intelligent Pathology Stereoscopic Analyzer system. These precise thresholds may be influenced by sample composition, event numbers, tissue fixation, IHC staining protocols, scanning parameters, image quality, ROI selection, and the AI quantification platform. Therefore, thresholds such as CD3 25.60%, CD3 32.41%, and CD21 56.53% should not currently be regarded as universally applicable clinical cut-offs. Third, overfitting remains an important concern. Although repeated random resampling, three-fold cross-validation, bootstrap analysis, sensitivity analyses, and homogeneous-treatment subgroup analyses were performed to assess model robustness, these internal validation methods cannot fully eliminate overfitting risk or substitute for independent external validation. In particular, the CD3-revised MZL-IPI model was less stable than the CD21-revised MZL-IPI in subgroup analyses, suggesting that it may be more susceptible to cohort-specific effects and potential overfitting. Accordingly, the revised MZL-IPI model proposed in this study should be considered exploratory and hypothesis-generating rather than a tool ready for immediate routine clinical use. Future multicenter studies based on larger independent cohorts and standardized tissue-processing, IHC-staining, digital-scanning, and AI-analysis workflows are needed to validate the reproducibility, calibration, generalizability, and clinical utility of this model.

## 5. Conclusions

In this study, AI-assisted quantitative IHC analysis identified CD3 and CD21 as markers associated with transformation risk and survival outcomes in MZL. CD3 may reflect T-cell infiltration and was associated with histologic transformation and PFS, whereas CD21 may reflect FDC network architecture and was independently associated with OS. Incorporating these markers into the MZL-IPI improved prognostic discrimination in this cohort, with the CD21-revised model showing more stable performance. Given the retrospective single-center design, limited AI-IHC analysis cohort, potential overfitting risk, platform dependency, and lack of external validation, the revised model should currently be regarded as an exploratory framework. Larger multicenter studies are required to validate its stability, reproducibility, and clinical utility before routine clinical application.

## Data Availability

The data presented in this study are available on request from the corresponding author due to ethical restrictions.

## Supplementary Materials

The following supporting information can be downloaded at https://www.mdpi.com/article/10.3390/diagnostics16101456/s1: Supplementary Figure S1: Distribution of expression levels of 13 AI-quantified IHC markers. Supplementary Figure S2: Expression differences in AI-quantified IHC markers across clinical subgroups. Supplementary Figure S3: Sensitivity analysis of the CD3-revised MZL-IPI model. Supplementary Table S1: Detailed Information for Patients with Marginal Zone Lymphoma in This Study. Supplementary Table S2: Summary of Clinical Data in This Study. Supplementary Table S3: Mean and Confidence Intervals of Immunohistochemical Marker Quantitative Values. Supplementary Table S4: Statistical results from differential and correlation analyses. Supplementary Table S5: Statistical results from survival analysis using log-rank test. Supplementary Table S6: Statistical results from differential analyses between MALT and IOI.
