# Supplementary material for: AI-Enhanced Quantitative IHC Analysis for Prognostic Stratification in Marginal Zone Lymphoma: Development of a Revised MZL-IPI Model
Source: Diagnostics (Basel). 2026 May 11;16(10):1456. doi: 10.3390/diagnostics16101456 (PMC13205237; doi:10.3390/diagnostics16101456)
Supplement: Supplementary file 1 [file diagnostics-16-01456-s001.zip › Supplementary figures.pdf]

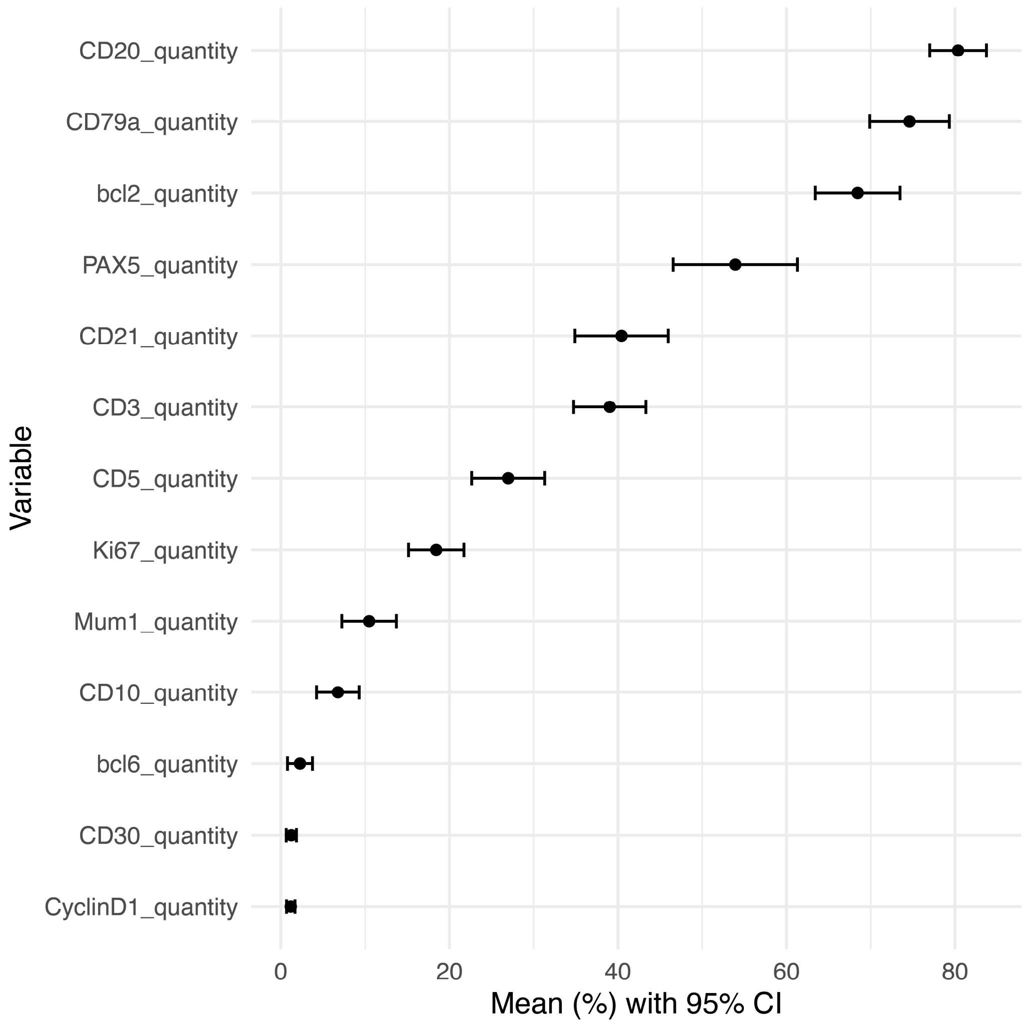

Supplementary Figure S1. Distribution of expression levels of 13 AI-quantified IHC markers.

The forest plot shows the mean and 95% confidence interval for each IHC marker, calculated as mean ± 1.96 × standard error.

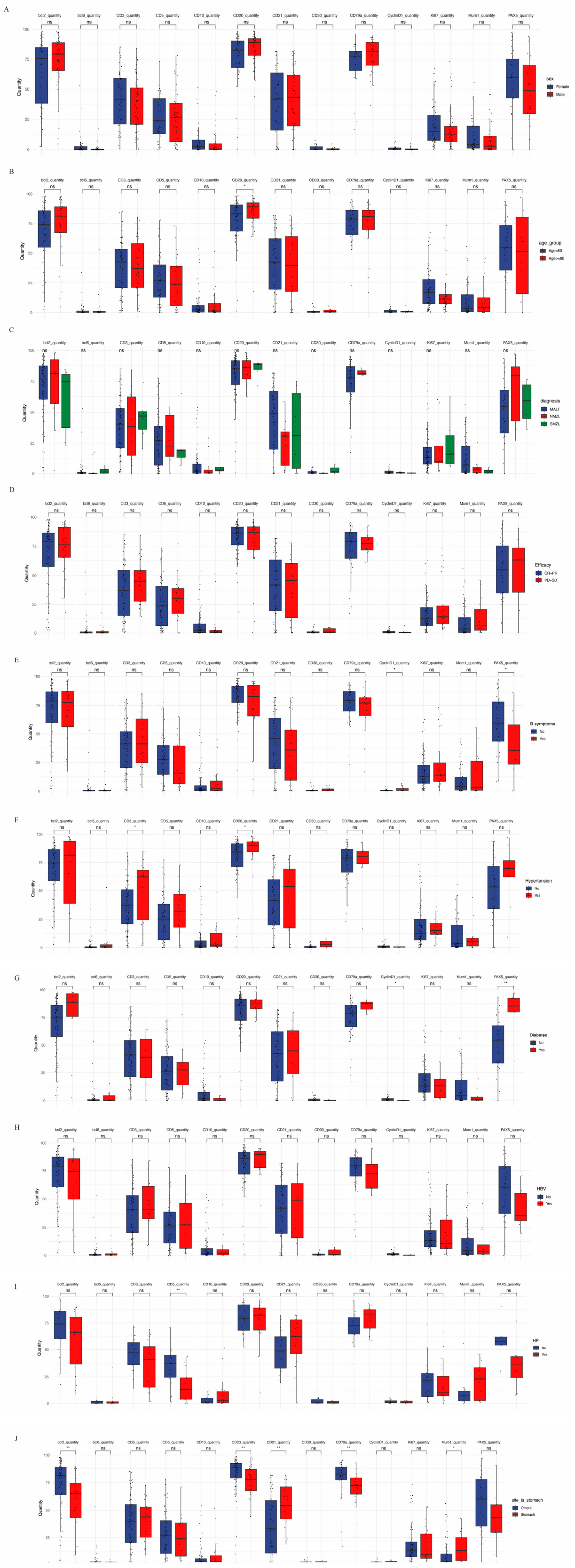

Supplementary Figure S2. Expression differences in AI-quantified IHC markers across clinical subgroups.

This figure consists of 10 faceted boxplots, each corresponding to the comparison of 13 IHC markers according to one binary clinical variable. **P* < 0.05; ***P* < 0.01.

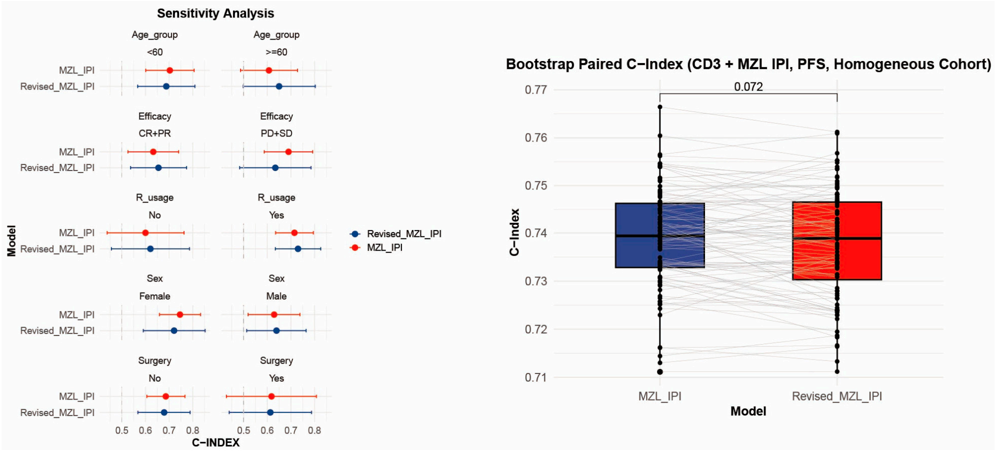

Supplementary Figure S3. Sensitivity analysis of the CD3-revised MZL-IPI model. The left panel shows a forest plot comparing the C-index and confidence intervals of the CD3-revised MZL-IPI and original MZL-IPI for predicting PFS across different clinical strata; the right panel compares the C-index of the CD3-revised MZL-IPI and original MZL-IPI in the homogeneous-treatment subgroup.
